# Supplementary material for: The alternative regenerative strategy of bearded dragon unveils the key processes underlying vertebrate tooth renewal
Source: eLife. 2019 Aug 16;8:e47702. doi: 10.7554/eLife.47702 (PMC6744223; doi:10.7554/eLife.47702)
Supplement: Figure 3—source data 1. — Absolute means of BrdU/PCNA-double positive cells in the entire dental epithelium (all regions) or relative amounts of BrdU/PCNA-double positive cells in the individual tooth regions (subdivisions I-IV, see Figure 3B) of pleurodont and acrodont teeth at d0 and d58, n = 3 biological replicates per group. [file elife-47702-fig3-data1.pdf]

		Absolute mean BrdU+/PCNA+ counts		Relative BrdU+/PCNA+ cell amounts per tooth region (I,II,III,IV)							
		Pleurodont	Acrodont	Pleurodont				Acrodont			
Time point	Replicate	All regions	All regions	I	II	III	IV	I	II	III	IV
d0	1	69,75	141,5	0,26957	0,16232	0,1913	0,37681	0,26626	0,2292	0,23828	0,26626
	2	100,5714286	83	0,2847	0,28266	0,34796	0,08469	0,34571	0,15968	0,23564	0,2589
	3	73,33333333	42,75	0,287	0,07289	0,43962	0,20045	0,51962	0,04902	0,09804	0,33334
d58	1	5,43	2,00	0,49206	0,1746	0,2381	0,09524	0,6993	0,0999	0,04995	0,14985
	2	10,38	7,75	0,36127	0,15655	0,40944	0,07225	0,22581	0,06452	0,22581	0,48387
	3	10,13	6,00	0,33317	0,20977	0,33317	0,1234	0,25	0,20833	0,375	0,16667
