# Supplementary material for: The alternative regenerative strategy of bearded dragon unveils the key processes underlying vertebrate tooth renewal
Source: eLife. 2019 Aug 16;8:e47702. doi: 10.7554/eLife.47702 (PMC6744223; doi:10.7554/eLife.47702)
Supplement: Figure 4—source data 1. — Total cell number (total cells), number of PCNA-positive cells (PCNA+ cells) and proportion of PCNA-positive cells in the DL/SDL region of one-week dental tissue cultures with intact (control) or removed (removal) OE, n = 4 biological replicates per group. [file elife-47702-fig4-data1.pdf]

	Replicate	Total cells (DAPI)	PCNA+ cells	Proportion of PCNA+/total cells
Control	1	119	69	0,579831933
	2	90	33	0,366666667
	3	106	66	0,622641509
	4	134	76	0,567164179
Removal	1	82	13	0,158536585
	2	112	37	0,330357143
	3	81	39	0,481481481
	4	204	45	0,220588235
