# Supplementary material for: The alternative regenerative strategy of bearded dragon unveils the key processes underlying vertebrate tooth renewal
Source: eLife. 2019 Aug 16;8:e47702. doi: 10.7554/eLife.47702 (PMC6744223; doi:10.7554/eLife.47702)
Supplement: Figure 5—source data 1. — ΔCt values in quantitative PCR for ALX1, SIX3, ISL1, FOXI1, and BARX1 genes in dental tissues from pleurodont and acrodont teeth, n = 3 biological replicates per gene. [file elife-47702-fig5-data1.pdf]

		ΔCt (Gene-*ACTB*)				
	**Replicate**	***ALX1***	***SIX3***	***ISL1***	***FOXI1***	***BARX1***
**Pleurodont**	**1**	4,09	6,67	7,02	13,88	6,87
	**2**	4,01	8,15	6,78	13,46	7,34
	**3**	4,29	8,17	6,83	13,37	8,03
**Acrodont**	**1**	8,40	10,84	9,22	9,54	6,64
	**2**	8,72	11,65	9,76	10,71	5,98
	**3**	7,65	11,22	10,05	9,70	7,26
